# Cultivar Variation in Hormonal Balance Is a Significant Determinant of Disease Susceptibility to *Xanthomonas campestris* pv. *campestris* in *Brassica napus*

**DOI:** 10.3389/fpls.2017.02121

**Published:** 2017-12-12

**Authors:** Md. Tabibul Islam, Bok-Rye Lee, Sang-Hyun Park, Van Hien La, Dong-Won Bae, Tae-Hwan Kim

**Affiliations:** ^1^Department of Animal Science, Institute of Agricultural Science and Technology, College of Agriculture and Life Science, Chonnam National University, Gwangju, South Korea; ^2^Biotechnology Research Institute, Chonnam National University, Gwangju, South Korea; ^3^Central Instrument Facility, Gyeongsang National University, Jinju, South Korea

**Keywords:** *Brassica napus*, disease susceptibility, hormonal balance, phenylpropanoid, redox, *Xanthomonas campestris* pv. *campestris*

## Abstract

This study aimed to directly elucidate cultivar variation in disease susceptibility and disease responses in relation to hormonal status in the interaction of *Brassica napus* cultivars and *Xanthomonas campestris* pv. *campestris* (Xcc), the causal agent of black rot disease. Fully expanded leaves of six *B. napus* cultivars (cvs. Capitol, Youngsan, Saturnin, Colosse, Tamra, and Mosa) were inoculated with Xcc. At 14 days post-inoculation with Xcc, cultivar variation in susceptibility or resistance was interpreted with defense responses as estimated by redox status, defensive metabolites, and expression of phenylpropanoid synthesis-related genes in relation to endogenous hormonal status. Disease susceptibility of six cultivars was distinguished by necrotic lesions in the Xcc-inoculated leaves and characterized concurrently based on the higher increase in reactive oxygen species and lipid peroxidation. Among these cultivars, as the susceptibility was higher, the ratios of abscisic acid (ABA)/jasmonic acid (JA) and salicylic acid (SA)/JA tended to increase with enhanced expression of SA signaling regulatory gene *NPR1* and transcriptional factor *TGA1* and antagonistic suppression of JA-regulated gene *PDF 1.2*. In the resistant cultivar (cv. Capitol), accumulation of defensive metabolites with enhanced expression of genes involved in flavonoids (chalcone synthase), proanthocyanidins (anthocyanidin reductase), and hydroxycinnamic acids (ferulate-5-hydroxylase) biosynthesis and higher redox status were observed, whereas the opposite results were obtained for susceptible cultivars (cvs. Mosa and Tamra). These results clearly indicate that cultivar variation in susceptibility to infection by Xcc was determined by enhanced alteration of the SA/JA ratio, as a negative regulator of redox status and phenylpropanoid synthesis in the *Brasica napus*–Xcc pathosystem.

## Introduction

Oilseed rape (*Brassica napus* L.), grown for the production of vegetable oil, animal feeds, and alternative fuel, is one of the major agro-economic crops. *Xanthomonas campestris* pv. *campestris* (Xcc), the causal agent of black rot, has become a major threat to *Brassica* species (Velasco et al., 2013). Disease symptoms caused by Xcc infection include V-shaped necrotic lesions on leaves and darkening of vascular tissues with extensive necrosis and chlorosis (Aires et al., 2011). Pathogen invasion induces different plant–pathogen defense reactions, including susceptibility, resistance, or non-host reactions (O’Donnell et al., 2003; Aires et al., 2011). One of the earliest physiological responses to pathogen infection is rapid reactive oxygen species (ROS) production (Venisse et al., 2001). ROS can reduce pathogen viability via direct antibacterial activity, and is also implicated in the oxidative damage of challenged plant cells through lipid peroxidation (Lee et al., 2007, 2013). ROS-scavenging systems are highly activated in resistance mechanisms, to scavenge the ROS and reduce oxidative stress. Glutathione (GSH) is one of the major non-enzymatic antioxidants present in a plant cell, which maintains the intracellular redox homeostasis by reducing cellular disulfide bonds (Finiti et al., 2014).

Over the recent decades, the roles of several phytochemicals, including secondary metabolites, in plant defense systems have been evaluated. Glucosinolates and its subsequent hydrolysis products play a role in the constitutive resistance of *Brassicaceae* to Xcc (Aires et al., 2011; Velasco et al., 2013). Plant phenolics are also involved in resistance against different plant diseases. In an integrated metabolo-proteomic study (Gunnaiah et al., 2012), metabolites of the phenylpropanoid pathway, such as hydroxycinnamic acid amides, phenolic glucosides, and flavonoids, were shown to be involved in resistance against *Fusarium graminearum*. Susceptibility of cabbage to Xcc infection also correlated with the decline in phenylalanine ammonia-lyase (PAL) activity and phenolic metabolite accumulation (Barman et al., 2015).

Plant hormones are major endogenous low molecular weight signal molecules involved in regulating mechanisms of resistance to pathogens. This regulation is achieved through the interplay of different signaling pathways, enabling each single hormone to assist or antagonize the others (Anderson et al., 2004; Sánchez-Vallet et al., 2012; Martínez-Medina et al., 2017). SA regulates the basal resistance and disease development in susceptible hosts (O’Donnell et al., 2003). In tomato–*Xanthomonas campestris* pv. *vesicatoria* (tomato–Xcv) interaction, ethylene (ET) synthesis is clearly dependent upon prior SA synthesis, and the removal of either hormone alters the course of symptom development, relative to the wild-type (O’Donnell et al., 2001). Several types of defense responses have been reported in Arabidopsis–*Xanthomonas* interactions. These defense responses include recognition of the pathogen, activation of signal transduction, and suppression of pathogen growth (Buell, 2002). A compatible interaction between SA-deficient mutant (*NahG*) of Arabidopsis and Xcc showed substantially more rapid bacterial growth and more disease progression than the wild-type (O’Donnell et al., 2003). ABA is usually involved in disease resistance mechanisms of various plant species (Mohr and Cahill, 2003), and mutually antagonistic interactions have been reported between ABA and ET (Anderson et al., 2004). Recently, JA- and SA-regulated defense pathways in *Trichoderma*-induced resistance to the root-knot nematode have been characterized (Martínez-Medina et al., 2017). In recent decades, a network of communication, referred to as “hormonal crosstalk,” among various hormone signaling pathways involved in pathogen resistance has been widely characterized by molecular studies based on experiments with mutant and transgenic lines (O’Donnell et al., 2001; Anderson et al., 2004; Sánchez-Vallet et al., 2012). However, the physiological significance of hormonal balance in disease resistance mechanisms has not been fully elucidated in host–pathogen interactions, especially in economic crops (Martínez-Medina et al., 2017).

The present study focused on evaluation of varietal differences in susceptibility or resistance responses and the endogenous hormonal status upon infection by the pathogen Xcc. We used six cultivars of *B. napus*, which are most widely grown. We tested the hypothesis that shifting of the endogenous hormonal balance caused by *Xcc* inoculation modulates the susceptible-to-resistant responses of the host plant, leading to genotypic variation in disease susceptibility.

## Materials and Methods

### Plant Culture and Pathogen Inoculation

Surface-sterilized seeds of six oilseed rape (*B. napus*) cultivars (Capitol, Youngsan, Saturnin, Colosse, Tamra, and Mosa) were grown in pots (0.6 l). When the seedlings had grown up to the four-leaf stage, they were divided into two groups, i.e., the control and the one with pathogen inoculation. The pathogenic bacterial (*X. campestris* pv. *campestris*, Xcc) strain (KACC No-10377) was obtained from the Korean Agricultural Culture Collection. Bacterium inoculum was cultured in Yeast Dextrose Calcium Carbonate (YDC) agar plate for 48 h at 30°C, and then the bacterial cells were scraped from plates and adjusted to a concentration of 10^8^ CFU/ml (0.2 OD A600 nm) with 0.85% NaCl solution. The inoculation process was followed by clipping of the leaf edges near the veins using mouth–tooth forceps. For every inoculation, the forceps was dipped into the bacterial suspension; the four youngest fully expanded leaves were inoculated. Fourteen days after inoculation, the leaves of Xcc non-inoculated (control) or Xcc-inoculated plants were, respectively, collected for the evaluation of different biochemical defense markers; they were immediately frozen in liquid nitrogen (N) and stored in a deep freezer (-80°C) for further analysis.

### Bacterial Populations

Two leaf discs from distinct infiltrated leaves of different inoculated plants were sampled at 14 days after inoculation and were homogenized in 200 μl sterile water. Serial dilutions of the homogenates were performed and 10 μl drops were spotted in triplicate for each dilution on plates supplemented with appropriate antibiotics. The plates were incubated at 30°C for 48 h and colonies were counted in spots containing 1–30 colonies (Xu et al., 2008).

### Determination of ROS, and Lipid Peroxidation Content

The H_2_O_2_ level was measured as described by Lee et al. (2007). To determine the H_2_O_2_ levels, the extracted solution was mixed with 0.1% titanium chloride in 20% (v/v) H_2_SO_4_, and the mixture was then centrifuged at 10,000 × *g* for 5 min. The absorbance of the supernatant was measured at 410 nm. The H_2_O_2_ level was calculated using the extinction coefficient of 0.28 μmol^-1^ cm^-1^.

For the visualization *in situ* of superoxide anion radical ($O_{2}^{•–}$), leaf discs were immersed in 0.1% solution of nitroblue tetrazolium (NBT) in K-phosphate buffer (pH 6.4), containing 10 mM Na-azide, and were vacuum-infiltrated for 60 min and illuminated until the appearance of dark spots, characteristic of the blue formazan precipitate. After bleaching in boiling ethanol, the leaf samples were photographed under a light microscope (Leica DM4000; Leica, Wetzlar, Germany) at 40× magnifications (Muneer et al., 2013).

The lipid peroxidation level was determined by measuring the concentration of malondialdehyde (MDA), as described previously (Lee et al., 2007).

### Phytohormone Analysis

Quantitative analysis of JA, SA, and ABA in the leaf tissue was performed according to Pan et al. (2010). JA, SA, and ABA extracts from 50 mg of well-ground leaves were injected into a reverse phase C18 Gemini high-performance liquid chromatography (HPLC) column for HPLC–electrospray ionization tandem mass spectrometry (HPLC–ESI–MS/MS) analysis. Agilent 1100 HPLC (Agilent Technologies), Waters C18 column (150 × 2.1 mm, 5 μm), and API3000 MSMRM (Applied Biosystems) were used for the analysis.

### Determination of Defensive Metabolites in the Phenylpropanoid Pathways

#### Total Phenolic and Flavonoid Contents

Total phenolic content in the leaves was determined by the Folin–Ciocalteu reagent assay (Lee et al., 2007). Total phenolic content was expressed as milligrams of gallic acid per gram fresh weight (FW). Total flavonoid content was measured by the aluminum chloride colorimetric assay (Zhishen et al., 1999) as expressed by milligrams of quercetin per gram FW.

#### Soluble and Insoluble Tannin Contents

Soluble and insoluble tannin contents were spectrophotometrically determined by the F–D method (Bubba et al., 2009). To determine the soluble and insoluble tannin content in 3.1 ml of ultrapure water, 0.1 ml of the extract and 300 μl of F–D reagent were added. After 3 min, 300 μl of a saturated aqueous solution of sodium carbonate was added. The results are expressed as milligrams of gallic acid per gram FW.

#### Proanthocyanidin and Total Hydroxycinnamic Acid (THA) Contents

The total proanthocyanidin content was measured using the 4-dimethylaminocinnamaldehyde (DMAC) assay (Prior et al., 2010). Total proanthocyanidins were quantified as catechin equivalents using a catechin standard curve. Determination of total hydroxycinnamic acid (THA) was performed by colorimetric methods using the chromogenic system of HCl-NaNO_2_–Na_2_MoO_4_–NaOH with chlorogenic acid standard (Štefan et al., 2014).

#### Glutathione and NADPH Redox

Total GSH and oxidized GSH (GSSG) were assayed using 5,5-dithiobisnitrobenzoic acid (DTNB), according to the method of Rahman et al. (2006). For the determination of total GSH, the test solution was prepared by taking 2 μl of sample diluted with 31 μl of 100 mM K-PO_4_ buffer containing 6.3 mM EDTA, then added with 140 μl of NADPH (0.248 mg/ml) and 20 μl of DTNB (6 mM), followed by GSH reductase (0.5 U). The absorbance was immediately recorded at 412 nm using a microplate reader, and measurements were taken every 1 min for 4 min. The test solution for GSSG determination was prepared by taking 5 μl of sample diluted with 28 μl of 100 mM K-PO_4_ buffer containing 6.3 mM EDTA, then added 140 μl of NADPH (0.248 mg/ml) and 20 μl of DTNB (6 mM), then GSH reductase (0.5 U) was added. The absorbance was immediately recorded at 412 nm using a microplate reader, with measurements taken every 1 min for 4 min. Reduced GSH and oxidized GSSG were quantified using respective standard curves and expressed as nanomoles per gram FW. The resulting reduced/oxidized GSH/GSSG ratio was calculated.

For the determination of NADPH and NADP^+^ concentration, fresh leaf samples (0.2 g) were immediately homogenized with 0.8 ml of 0.2 M NaOH for the NADPH assay and with 0.2 N HCl for the NADP^+^ assay. The supernatants following a centrifugation 12,000 × *g* for 10 min at 4°C were heated at 95°C for 1 min and stopped in ice-bath. The supernatant for NADP^+^ determination was adjusted to pH 5–6 by 0.2 M NaOH or to pH 7–8 with 0.2 N HCl. The oxidized and reduced pyridine nucleotide contents were determined using the protocol of Queval and Noctor (2007).

### Isolation of Total RNA and Quantitative Real-Time PCR

Total RNA was isolated from 200 mg leaf tissue using the SV Total RNA Isolation System (Promega). First-strand cDNAs were synthesized using the GoScript Reverse Transcription System (Promega). The gene expression level was quantified on a light cycler real-time PCR detection system (Bio-Rad) with SYBR Premix Ex TaqTM (TaKaRa, DALIAN). The sequences of primers are presented in Supplementary Table S1. All the quantifications were normalized to ACTIN. The qRT-PCR reactions were performed in triplicates for each of the three independent samples. Quantification of the relative transcript level was performed using the 2^-ΔΔC_t_^ method (Livak and Schmittgen, 2001).

### Statistical Analysis

A completely randomized design was used with three replicates for six cultivars and two pathogen inoculation treatments. An individual pot containing three plants represented as a replicate. Student’s *t*-test was employed to compare the means of separate replicates by using software SAS (version 9.1) (SAS Institute Inc., Cary, NC, United States). Different letter in tables indicates statistically significant difference at *P* < 0.05. For principal component analysis (PCA) all biochemical defenses markers were considered in Xcc-inoculated plants of six different cultivars of *B. napus*. PCA analysis was performed using the Factor analysis and data mining with R (FactoMineR) package (Husson et al., 2008).

## Results

### Disease Symptoms, Bacterial Populations, and Oxidative Stress Development

**FIGURE 1:**
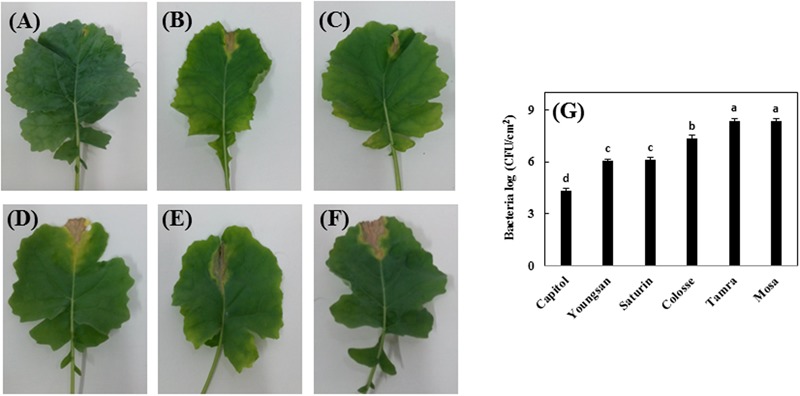
Development of necrotic lesions in leaves of *Brassica napus* cultivars inoculated by *Xanthomonas campestris* pv. *campestris* (Xcc), which causes the black rot disease; **(A)** Capitol, **(B)** Youngsan, **(C)** Saturnin, **(D)** Colosse, **(E)** Tamra, and **(F)** Mosa. Bacterial populations in the inoculated areas were measured 14 days after inoculation and expressed as log of colony-forming units per square cm (cfu/cm^2^). **(G)** Statistical groups were identified using Duncan’s multiple range test. Different letters indicate significant differences at *P* < 0.05.

*Xanthomonas campestris* pv. *campestris*-inoculation induced V-shaped necrosis in leaf margins. Among the six cultivars, cv. Capitol showed least symptoms (**Figure 1A**), whereas severe necrosis occurred in cvs. Colosse, Tamra, and Mosa (**Figures 1D–F**) and moderate symptoms were observed in cvs. Youngsan and Saturnin (**Figures 1B,C**). The lowest bacterial populations were measured in cv. Capitol, while the highest bacterial populations were observed in cvs. Tamra and Mosa (**Figure 1G**).

**FIGURE 2:**
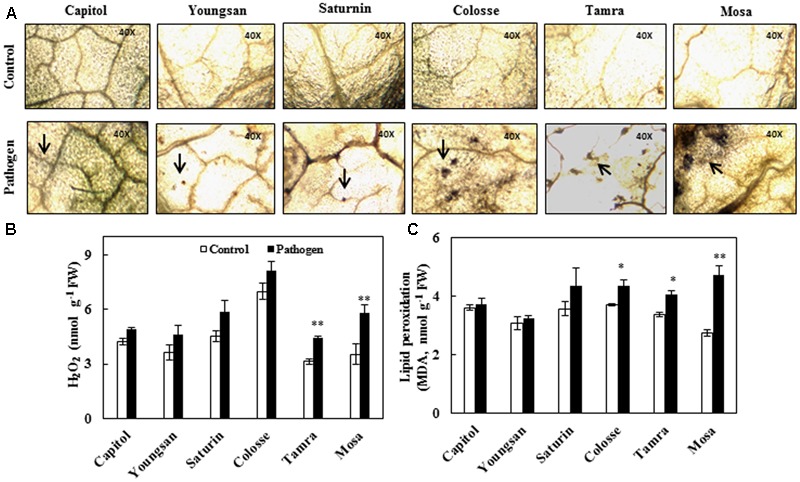
Oxidative stress development in control (open bar) and *X. campestris* pv. *campestris* (Xcc) inoculated (filled bar) leaves of six *B. napus* cultivars; **(A)** Superoxide anion radical ($O_{2}^{•–}$) accumulation visualized by nitroblue tetrazolium (NBT) staining, **(B)** hydrogen peroxide (H_2_O_2_), and **(C)** lipid peroxidation (malondialdehyde, MDA). Data are presented as means ± SE for *n* = 3. Asterisks indicate significant differences between the control and pathogen-stressed plants; ^∗^*P* < 0.05, ^∗∗^*P* < 0.01, ^∗∗∗^*P* < 0.001.

Reactive oxygen species production was determined by visualizing superoxide anion radical ($O_{2}^{•–}$) accumulation and quantifying hydrogen peroxide (H_2_O_2_) content. Relatively higher accumulation of superoxide anion radical occurred in cvs. Colosse, Tamra, and Mosa (**Figure 2A**). The increase in H_2_O_2_ content caused by Xcc-inoculation was also significant in cvs. Tamra (+41%) and Mosa (+63%) (**Figure 2B**). Significant increase in lipid peroxidation level, as determined by MDA content, was observed in cvs. Colosse (+17%), Tamra (+20%), and Mosa (+72%). The lowest changes in ROS production and lipid peroxidation were observed in cv. Capitol (**Figure 2**).

### Phytohormone Contents

**FIGURE 3:**
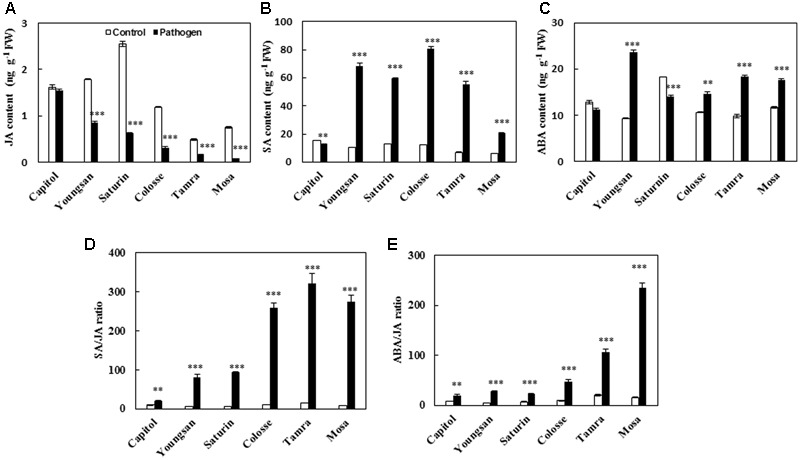
Phytohormonal changes in response to *X. campestris* pv. *campestris* (Xcc) inoculation in six different *B. napus* cultivars; **(A)** Jasmonic acid (JA), **(B)** salicylic acid (SA), **(C)** abscisic acid (ABA), **(D)** ratio of SA/JA, and **(E)** ratio of ABA/JA. Data are presented as means ± SE for *n* = 3. Asterisks indicate significant differences between the control and pathogen-stressed plants; ^∗^*P* < 0.05, ^∗∗^*P* < 0.01, ^∗∗∗^*P* < 0.001.

*Xanthomonas campestris* pv. *campestris*-inoculation decreased the endogenous level of JA in all cultivars examined, with a reduction ranging from -4.8% (cv. Capitol) to -89.3% (cv. Mosa) compared with the non-pathogen inoculated control (**Figure 3A**). SA was significantly increased in all cultivars except cv. Capitol (**Figure 3B**). Similarly, ABA was also increased in all cultivars except cvs. Capitol and Saturin (**Figure 3C**). The resulting SA/JA and ABA/JA ratios increased significantly in all cultivars examined, with the highest increase observed in cv. Mosa (33.1- and 14.0-fold, respectively) compared to the control (**Figures 3D,E**).

### Phytohormone-Signaling-Related Gene Expression

**FIGURE 4:**
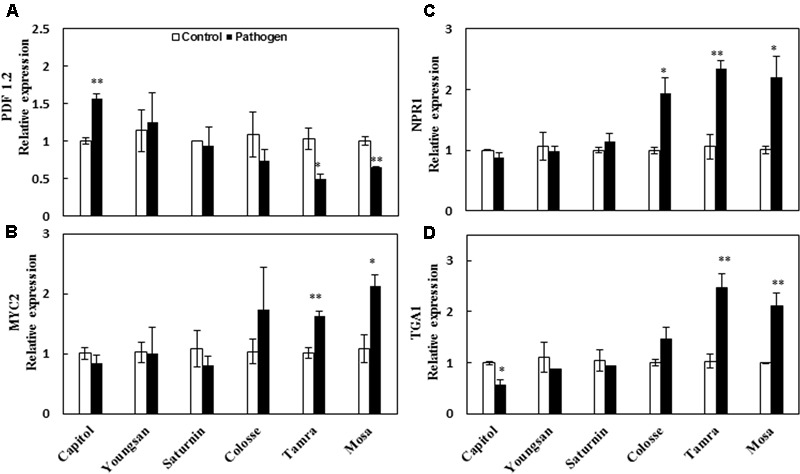
Relative expression of phytohormone-signaling regulated genes in the control (open bar) and *X. campestris* pv. *campestris* (Xcc) inoculated (filled bar) leaves of six different *B. napus* cultivars. **(A)** Jasmonic acid-regulated gene *PDF 1.2*, **(B)** ABA-regulated gene *MYC2*, **(C)** salicylic acid-regulated gene *NPR1*, and **(D)** *TGA1*. Data are presented as means ± SE for *n* = 3. Asterisks indicate significant differences between the control and pathogen-stressed plants; ^∗^*P* < 0.05, ^∗∗^*P* < 0.01, ^∗∗∗^*P* < 0.001.

The relative expression of a JA-responsive gene, *Plant defensin 1.2* (*PDF 1.2*), was enhanced significantly by Xcc-inoculation only in cv. Capitol (+56%). However, *PDF 1.2* expression was depressed in cvs. Tamra (-53%) and Mosa (-36%) (**Figure 4A**). Expression of the transcriptional factor *MYC2*, an ABA-signaling regulatory gene, was significantly increased in cvs. Tamra (+60%) and Mosa (+97%) (**Figure 4B**). The SA-regulated gene *NPR1* was upregulated in cvs. Colosse (+94%), Mosa (+120%), and Tamra (+121%) (**Figure 4C**). Similarly, expression of the transcriptional factor *TGA1*, which also regulates the SA-signaling pathway, was enhanced in cvs. Tamra (+139%) and Mosa (+112%), while depressed in cv. Capitol (-44%) (**Figure 4D**).

### NADPH and Glutathione Redox Status

**FIGURE 5:**
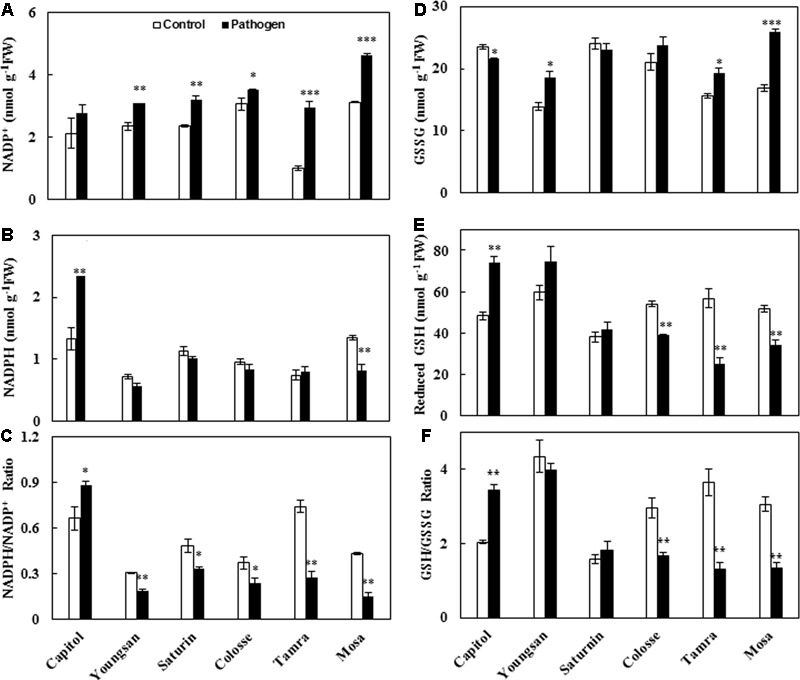
Glutathione and NADPH redox responses to *X. campestris* pv. *campestris* (Xcc) inoculation in six different *B. napus* cultivars: **(A)** NADP^+^, **(B)** NADPH, **(C)** the NADP^+^/NADPH ratio, **(D)** oxidized glutathione (GSSG), **(E)** reduced glutathione (GSH) content, and **(F)** the ratio of GSH to GSSG. Data are presented as means ± SE for *n* = 3. Asterisks indicate significant differences between the control and pathogen-stressed plants; ^∗^*P* < 0.05, ^∗∗^*P* < 0.01, ^∗∗∗^*P* < 0.001.

*Xanthomonas campestris* pv. *campestris*-inoculation significantly increased NADP^+^ concentration in all cultivars except cv. Capitol (**Figure 5A**). A significant increase in NADPH concentration was observed only in cv. Capitol (+76%), but a decrease was observed in cv. Mosa (-40%) (**Figure 5B**). The resulting NADPH/NADP^+^ ratio was significantly increased by Xcc-inoculation in cv. Capitol, while it was decreased more distinctly in cvs. Tamra (-63.0%) and Mosa (-64.7%) (**Figure 5C**).

Similarly, the oxidized GSH, GSH disulfide (GSSG), was significantly increased in cvs. Tamra (+23%) and Mosa (+53%), while it was decreased in cv. Capitol (-6.3%) (**Figure 5D**). Opposite responses to Xcc-inoculation were observed for reduced GSH content (**Figure 5E**). The resulting reduced/oxidized GSH/GSSG ratio increased in cv. Capitol (+68%), whereas it significantly decreased in cvs. Colosse (-44%), Tamra (-72%), and Mosa (-36%) (**Figure 5F**).

### Contents of Defensive Metabolites of Phenylpropanoid Pathway

In cv. Capitol, Xcc-inoculation significantly increased total phenolic (+18.7%), flavonoids (+18.5%), total tannin (+32.3%), proanthocyanidin (90.5%), and THA (+29.7%), whereas these phenolic compounds were not significantly changed or slightly decreased in other cultivars. The overall content of these compounds in Xcc-inoculated plants was higher in cv. Capitol compared with other cultivars (**Table 1**).

**Table 1:** Changes in the contents of phenylpropanoid compounds as affected by *Xanthomonas campestris* pv. *campestris* (Xcc) inoculation in six different *Brassica napus* cultivars.

Secondary metabolite content (mg g^-1^ FW)	Treatment	Different cultivar of *B. napus*					
		Capitol	Youngsan	Saturin	Colosse	Tamra	Mosa
Total phenolic	Control	0.91 ± 0.03^b^	0.88 ± 0.03^b^	0.95 ± 0.06^a^	1.00 ± 0.04^a^	0.96 ± 0.03^a^	0.97 ± 0.01^a^
	Xcc	1.08 ± 0.04^a^	0.96 ± 0.01^a^	0.89 ± 0.03^a^	1.14 ± 0.05^a^	1.01 ± 0.02^a^	1.09 ± 0.05^a^
Total flavonoids	Control	4.65 ± 0.15^b^	4.10 ± 0.32^a^	4.69 ± 0.58^a^	5.26 ± 0.27^a^	5.21 ± 0.06^a^	4.34 ± 0.29^a^
	Xcc	5.51 ± 0.18^a^	4.99 ± 0.59^a^	4.27 ± 0.32^a^	5.78 ± 0.61^a^	5.49 ± 0.20^a^	5.34 ± 0.26^a^
Soluble tannin	Control	0.74 ± 0.02^b^	0.75 ± 0.01^b^	0.72 ± 0.01^a^	0.77 ± 0.01^a^	0.88 ± 0.01^a^	0.80 ± 0.01^a^
	Xcc	0.89 ± 0.04^a^	0.79 ± 0.01^a^	0.76 ± 0.03^a^	0.87 ± 0.10^a^	0.84 ± 0.01^b^	0.86 ± 0.03^a^
Insoluble tannin	Control	3.61 ± 0.29^b^	3.95 ± 0.29^a^	3.79 ± 0.01^a^	3.54 ± 0.32^a^	3.40 ± 0.16^a^	4.39 ± 0.23^a^
	Xcc	5.22 ± 0.23^a^	4.05 ± 0.17^a^	4.21 ± 0.53^a^	4.05 ± 0.18^a^	3.43 ± 0.20^a^	4.82 ± 0.27^a^
Proanthocyanidin	Control	1.26 ± 0.10^b^	1.75 ± 0.39^a^	1.98 ± 0.33^a^	1.68 ± 0.43^a^	1.83 ± 0.10^a^	2.19 ± 0.01^a^
	Xcc	2.40 ± 0.25^a^	2.41 ± 0.17^a^	2.08 ± 0.35^a^	1.60 ± 0.29^a^	1.12 ± 0.23^b^	2.03 ± 0.35^a^
Total hydroxycinnamic acid	Control	0.64 ± 0.03^b^	0.61 ± 0.01^a^	0.62 ± 0.02^a^	0.64 ± 0.11^a^	0.62 ± 0.07^a^	0.55 ± 0.01^a^
	Xcc	0.83 ± 0.04^a^	0.65 ± 0.02^a^	0.62 ± 0.12^a^	0.76 ± 0.04^a^	0.72 ± 0.03^a^	0.52 ± 0.04^a^

*Values are means of three biological replicates. Different letters in a vertical column indicate significant difference at *P* < 0.05.*

### Gene Expression Involved in Phenylpropanoid Pathway

**FIGURE 6:**
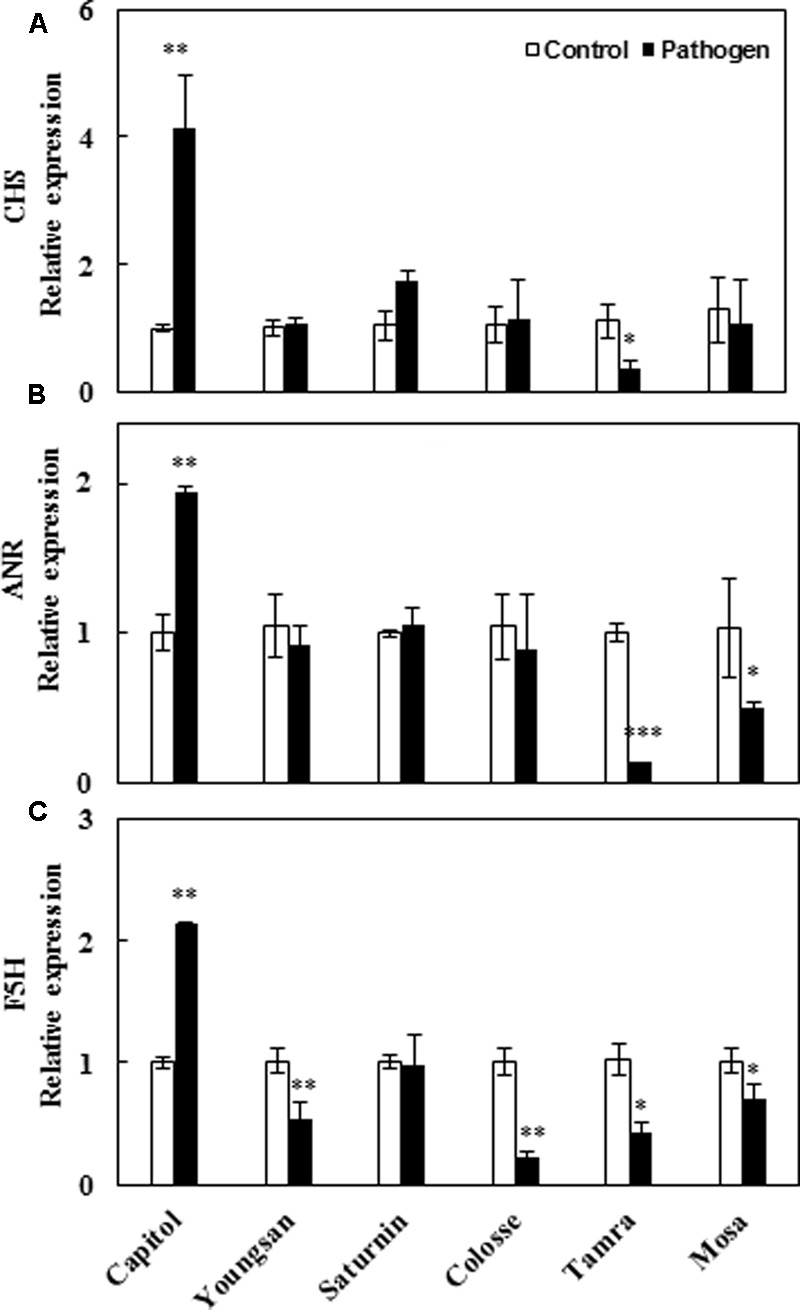
Relative expression of genes involved in phenylpropanoid synthesis pathway in control (open bar) and *X. campestris* pv. *campestris* (Xcc) inoculated (filled bar) leaves of six different *B. napus* cultivars: **(A)** *Chalchon synthase*, **(B)** *anthocyanidin reductase*, and **(C)** *ferulate-5-hydroxylase*. Data are presented as means ± SE for *n* = 3. Asterisks indicate significant differences between the control and pathogen-stressed plants; ^∗^*P* < 0.05, ^∗∗^*P* < 0.01, ^∗∗∗^*P* < 0.001.

To compare cultivar variation in polyphenol biosynthesis in response to Xcc-inoculation, relative expression of three major genes of the phenylpropanoid pathway [chalcone synthase (*CHS*), anthocyanidin reductase (*ANR*), and ferulate-5-hydroxylase (*F5H*) that regulate the synthesis of flavonoids, proanthocyanidins, and hydroxycinnamic acids, respectively] was evaluated. Xcc-inoculation significantly enhanced the expression of *CHS* (4.1-fold), *ANR* (1.9-fold), and *F5H* (2.1-fold) genes in cv. Capitol, whereas the expression of these genes was significantly depressed or not changed in other cultivars (**Figures 6A–C**).

### Principal Component Analysis

**FIGURE 7:**
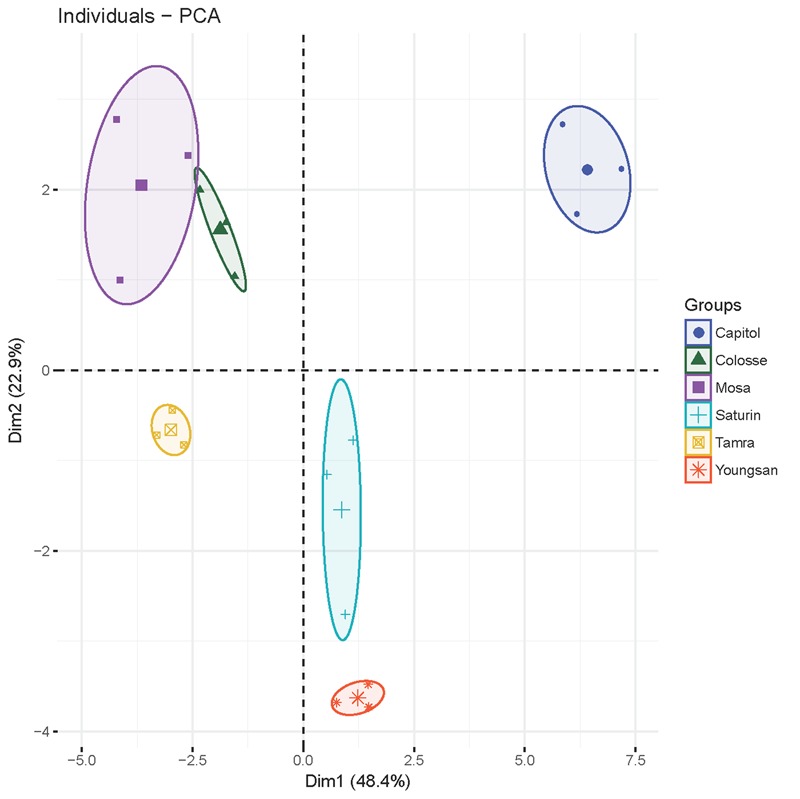
The plot of principal component analysis for *X. campestris* pv. *campestris* (Xcc)-inoculation-responsive changes of physiological and defensive parameters in six different *B. napus* cultivars.

Principal component analysis was applied to detect any possible clusters with respect to the responses of physiological and defensive parameters to Xcc*-*inoculation in six different cultivars (**Figure 7**). The cumulative contribution of the first and the second principal components attained 71.3%. Principal component 1 (PCA1) explained up to 48.4% of the total variance and principal component 2 (PCA2) explained 22.9% of the variation. PCA1 was highly contributed by JA (0.99), *PDF 1.2* (0.96), *NPR1* (-0.90), *TGA1* (-0.89), *MYC2* (-0.84), SA/JA ratio (-0.93), ABA/JA ratio (-0.74), MDA (-0.61), GSH/GSSG ratio (0.78), NADP^+^/NADPH ratio (0.84), proanthocyanidins (0.70), THA (0.74), insoluble tannin (0.57), *CHS* (0.89), *ANR* (0.94), and *F5H* (0.85). The second PCA separated the samples on the basis of soluble tannin (0.91), insoluble tannin (0.57), flavonoids (0.69), total phenolics (0.86), SA (-0.58), ABA (-0.65), and NADPH (0.54) values. Notably, all parameters of cv. Capitol were clustered into one group as they had highly positive correlation with PCA1 and PCA2, whereas cvs. parameters of cultivars Youngsan and Saturnin were clustered into another group as they had a positive correlation with PCA1 and a higher negative correlation with PCA2. Parameters of cvs. Colosse, Tamra, and Mosa were also clustered into another group; they had a negative correlation with PCA1.

## Discussion

Accumulation of ROS, such as $O_{2}^{•–}$, H_2_O_2_, and hydroxyl radical, and the induction of their scavenging enzymes are basic responses to plant stresses caused by a wide range of environmental stresses (Lee et al., 2009) and pathogen infection (Silva et al., 2004; Finiti et al., 2014). The dynamic balance between ROS and scavenging enzymes might be disturbed under a stressed condition. Rapid production of ROS leading oxidative burst is described as one of the earliest plant responses to pathogen infection. Due to the impairment of ROS-scavenging system, enhanced lipid peroxidation (MDA) occurs, which is associated with necrosis of plant tissues (Venisse et al., 2001). We previously defined cultivar variation in drought tolerance with regard to N use efficiency for N uptake and *de novo* protein synthesis (Lee et al., 2015), and sulfur (S) use efficiency in relation to photosynthetic activity (Lee et al., 2016) in *B. napus* cultivars. We suggested that cultivar differences in pathogen resistance exist. Therefore, in the present study, we elucidated *in vivo* regulation of the pathogen resistance mechanism. As expected, cultivar variation in disease symptom development, ROS production, and lipid peroxidation was observed following Xcc-inoculation. Varietal differences in the V-shaped necrotic lesion area and higher bacterial population (**Figure 1**) were characterized by the increased ROS accumulation and lipid peroxidation level upon Xcc-inoculation (**Figure 2**), which can be classified into three groups: the most symptomless, resistant (cv. Capitol), moderate (cvs. Youngsan, Saturnin, and Colosse), and susceptible (cvs. Tamra and Mosa). Elevated ROS level not only alters transcription of genes and metabolic pathway, but also is involved in the biosynthesis and functioning of phytohormones (Choudhury et al., 2017). A complex interplay between ROS and phytohormones has been recently elucidated under abiotic stress: ROS and auxin-mediated signaling (Xia et al., 2015), ABA-dependent ROS production (Zhou et al., 2014; Xia et al., 2015), and SA interaction with ROS (Herrera-Vásquez et al., 2015).

For hormonal regulation in the host plant–pathogen interaction, SA-dependent signaling is activated by biotrophic pathogens while JA-dependent signaling is activated by necrotrophic pathogens and leaf-chewing insects (Pieterse et al., 2009) for induction of plant defenses. Antagonistic hormonal interaction is involved in regulating defense responses (Anderson et al., 2004; Martínez-Medina et al., 2017). Among the six phytohormones analyzed in the present study, cultivar variation and Xcc-inoculation effects were significant only in JA, SA, and ABA levels. Overall Xcc-inoculation depressed JA level (**Figure 3A**), but enhanced SA level (except in cv. Capitol; **Figure 3B**) and ABA level (except in cvs. Capitol and Saturnin; **Figure 3C**). The SA/JA and ABA/JA ratios altered by Xcc-inoculation reflected cultivar variation in disease symptom development and ROS status, as shown by the lowest increase in both hormonal balances in cv. Capitol (the most symptomless cultivar) (**Figures 3D,E**) with enhanced expression of JA-regulated gene *PDF 1.2* (**Figure 4A**), but more distinct increases in the ratios with higher enhancement of *MYC2, NPR1*, and *TGA1* gene expressions in cvs. Tamra and Mosa (susceptible cultivars) (**Figures 4B–D**). These results indicate that JA-based resistance as well as SA- and/or ABA-associated susceptible responses occurred in *B. napus*–Xcc interaction. However, in the SA-deficient mutant (*NahG*) of Arabidopsis, severe necrosis with JA accumulation was observed 48 h after Xcc inoculation (O’Donnell et al., 2003). ABA application also showed more rapid proliferation of Xcc in Arabidopsis (Ho et al., 2013). The discrepancy of SA- and JA-dependent responses in *B. napus* (**Figures 3A,B**) and Arabidopsis, that needs to be elucidated further, might be associated with the differences in the necrotrophic phase and susceptibility to Xcc infection between the two host plants. Similarly, the lower JA level in JA-insensitive *coi1-1* mutants of Arabidopsis was responsible for susceptibility to the pathogen *Pythium irregulare* (Adie et al., 2007), and higher SA level was found in wheat mutants susceptible to *F. graminearum* in the later stage of infection (Ding et al., 2011). Martínez-Medina et al. (2017) have recently reported that shifting from priming of SA- to JA-regulated defenses induced resistance to root-knot nematode in tomato.

To investigate whether the alteration of JA-based hormonal balance by Xcc-inoculation, which reflected cultivar variation in disease symptom development, is a significant regulating factor of disease tolerance, we estimated the responses of redox status, defensive metabolites, and expression of genes involved in the phenylpropanoid synthesis pathway. NADPH is characterized as a cofactor in proline biosynthesis (Shinde et al., 2016), which is enhanced under stress conditions (Kim et al., 2004; Lee et al., 2013). NADPH concentration was significantly (*P* < 0.01) increased by Xcc-inoculation in cv. Capitol, while it was decreased in cv. Mosa (**Figure 5B**). The resulting ratio of NADPH/NADP^+^ was significantly increased only in cv. Capitol, while it was decreased in other five cultivars with the highest reduction in cvs. Tamra and Mosa (**Figure 5C**). GSH is an important non-enzymatic antioxidant in plant cells (Foyer and Noctor, 2011). Intracellular GSH redox homeostasis is accomplished by reducing the cellular disulphide bonds with a high intracellular concentration of GSH leading to an increase in GSH/GSSG ratio (Apel and Hirt, 2004). In the present study, Xcc-inoculation significantly (*P* < 0.01) enhanced GSH concentration in cv. Capitol, while decreased in cvs. Tamra and Mosa (**Figure 5E**). The resulting reduced/oxidized GSH/GSSG ratio showed the same pattern with GSH response to Xcc-inoculation (**Figure 5F**). The results of the present study suggest that NADP^+^ and the oxidized GSH form (GSSG) increased relatively higher in susceptible cultivars (cvs. Tamra and Mosa), leading to lower NADPH/NADP^+^ and GSH/GSSG ratios, which reflects an oxidized status. This agrees with cultivar variation in oxidative burst in relation to the alteration of SA/JA and ABA/JA ratios, which occurred in response to Xcc-inoculation. It thus concludes that the alteration of SA/JA and ABA/JA ratios is a defense response in alleviating oxidative imbalance caused by Xcc-infection. Indeed, we found that SA/JA and ABA/JA ratios were closely related (*P* < 0.01) with GSH/GSSG and NADPH/NADP^+^ ratios, respectively, in Xcc-inoculated plants of the six cultivars (**Table 2**). Similarly, hexanoic acid-induced GSH/GSSG alteration was an earlier defense response to reduce oxidative stress against *Botrytis cinerea* (Finiti et al., 2014) and reduced GSH/GSSG ratio was responsible for susceptibility to *B. cinerea* in tomato (Kuźniak and Sklodowska, 2005).

**Table 2:** Linear relationships between descriptive parameters of hormonal status [jasmonic acid (JA), abscisic acid (ABA), and salicylic acid (SA) signaling gene expressions and JA-based hormone ratio] and defense responses (redox and phenylpropanoid synthesis-related genes) as affected by *Xanthomonas campestris* pv. *campestris* (Xcc) inoculation in six different cultivars of *B. napus*.

	GSH/GSSG	NADPH/NADP^+^	*CHS*	*ANR*	*F5H*
*PDF 1.2*	*r* = 0.735^∗∗∗^	*r* = 0.533^∗^	*r* = 0.567^∗^	*r* = 0.753^∗∗∗^	*r* = 0.670^∗∗^
*MYC2*	*r* = -0.418	*r* = -0.421	*r* = -0.292	*r* = -0.289	*r* = -0.398
*NRP1*	*r* = -0.696^∗∗∗^	*r* = -0.498^∗^	*r* = -0.554^∗^	*r* = -0.645^∗∗^	*r* = -0.621^∗∗^
*TGA1*	*r* = -0.695^∗∗∗^	*r* = -0.511^∗^	*r* = -0.547^∗^	*r* = -0.734^∗∗∗^	*r* = -0.593^∗∗^
SA/JA	*r* = -0.792^∗∗∗^	*r* = -0.609^∗∗^	*r* = -0.654^∗∗^	*r* = -0.774^∗∗∗^	*r* = -0.702^∗∗∗^
ABA/JA	*r* = -0.582^∗∗^	*r* = -0.478^∗^	*r* = -0.403	*r* = -0.649^∗∗^	*r* = -0.330

*Asterisks indicate significant differences; ^∗^*P* < 0.05, ^∗∗^*P* < 0.01, ^∗∗∗^*P* < 0.001.*

Secondary metabolites that can be produced by various routes are involved in plant disease resistance (Iriti et al., 2005; Gunnaiah et al., 2012; Pieterse et al., 2012; Velasco et al., 2013). The results of the present study showed further accumulation of defensive metabolites of the phenylpropanoid pathway, such as flavonoids, hydroxycinnamic acids, and total phenolics in Xcc-inoculated plants of cv. Capitol (**Table 1**). However, these compounds were not changed or decreased in cvs. Tamra and Mosa. This indicates an alleviating effect of phenylpropanoid accumulation on resistance to Xcc in *B. napus* cultivars. Similarly, accumulation of glucosinolates and polyphenols (hydroxycinnamic acids and flavonoids) enhanced the resistance of *B. rapa* to Xcc (Velasco et al., 2013). In another study, resistance of wheat against *F. graminearum* was attributed to enhanced level of hydroxycinnamic acid amides, phenolic glucosides, and flavonoids (Gunnaiah et al., 2012). In addition, accumulation of proanthocyanidin conferred resistance against gray mold caused by *B. cinerea* in grapevine (Iriti et al., 2005).

We further interpreted the responses of genes involved in the phenylpropanoid synthesis pathway in relation to JA-based hormonal balance altered by Xcc-inoculation, which reflected cultivar variation in disease symptom development. The genes, *CHS, F5H*, and *ANR*, are involved in the biosynthetic pathways of flavonoids, hydroxycinnamic acids, and proanthocyanidins, respectively (Fornalé et al., 2015). In the present study, Xcc-inoculation significantly (*P* < 0.01) enhanced the expression of these three genes in cv. Capitol, while it depressed or did not affect their expression in cvs. Tamra and Mosa (**Figure 6**), showing a reversed pattern and cultivar variation in disease symptoms (**Figure 1**) and ROS production (**Figure 2**). To elucidate whether the altered hormonal status by Xcc-inoculation is involved in susceptibility and resistance responses to Xcc-inoculation, correlations among the expression of these three genes and of JA-, ABA-, and SA-regulated genes were analyzed. The JA-regulated gene *PDF 1.2* was positively correlated with the expression of *CHS* (*P* < 0.05), *ANR* (*P* < 0.001), and *F5H* (*P* < 0.01) genes, respectively (**Table 2**). This indicates that the highest JA level (**Figure 3A**) with enhanced *PDF 1.2* expression (**Figure 4A**) was a positive regulator of phenylpropanoid synthesis, resulting in elevated resistance in cultivar Capitol, but conversely in susceptibility in cultivars Tamra and Mosa. The JA signaling pathway regulates resistance against necrotrophic and hemibiotrophic pathogens by up-regulating the ERF branch marker gene *PDF 1.2* (Berrocal-Lobo et al., 2002). In the present study, the SA signaling regulatory gene *Non-expresor PR 1* (*NPR1*) and the transcriptional factor *TGA1* were up-regulated along with *MYC2*, a positive regulator of the ABA signaling pathway in susceptible cultivars (cvs. Tamra and Mosa) (**Figure 4**), with concurrent suppression of phenylpropanoid synthesis-related genes (**Figure 6**). Susceptible mutants of wheat to the hemibiotroph *F. graminearum* had higher SA content compared with the resistant genotype in the later stage of infection (Ding et al., 2011). Indeed, the SA signaling regulatory gene *NPR1* and transcriptional factor *TGA1* were negatively correlated with the expression of phenylpropanoid synthesis-related genes (**Table 2**). These results indicate activation of the SA-regulated defense signal with enhanced expression of *NPR1* and *TGA* in cvs. Tamra and Mosa (**Figures 4C,D**), and suppression of expression of the JA signaling pathway (Pieterse et al., 2012), leading to susceptibility to Xcc infection. Similarly, Spoel et al. (2007) reported that induction of the SA-signaling pathway by inoculation of avirulent *P. syringae* suppressed the JA signaling, rendering Arabidopsis susceptible to the necrotrophic fungus *Alternaria brassicola*. The present study also showed that enhanced alteration of the SA/JA ratio was responsible for susceptibility to Xcc infection (**Figure 3D**) in accordance with cultivar variation in symptom development (**Figure 1**) and ROS accumulation (**Figure 2**). Highly significant correlations between the SA/JA ratio with redox status [GSH/GSSG (*P* < 0.001) and NADPH/NADP^+^ (*P* < 0.01) ratios] and the expression of phenylpropanoid synthesis-related genes [*CHS* (*P* < 0.01), *ANR* (*P* < 0.001), and *F5H* (*P* < 0.01)] were observed (**Table 2**). In addition, PCA showed that cv. Capitol was positively correlated with PCA1 and PCA2 which were determined by JA-based hormonal balance, *PDF1.2* expression, phenolic metabolites, genes involved in the phenylpropanoid synthesis pathway, and the redox status.

Taken together, the results of the present study suggest that cultivar variation in disease susceptibility to Xcc-infection was determined by enhanced alteration of the SA/JA ratio, as a negative regulator of redox status and phenylpropanoid synthesis. To the best of our knowledge, the present study is the first to directly elucidate the physiological significance of hormonal balance in disease defense mechanisms, with regard to cultivar variation in disease susceptibility, especially in an economically important crop, *B. napus*.

## Author Contributions

MTI and T-HK designed the experiment and wrote the manuscript. MTI and B-RL carried out the experiment. T-HK, B-RL, S-HP, VHL, and D-WB participated in data interpretation and critical reading of the manuscript.

## Conflict of Interest Statement

The authors declare that the research was conducted in the absence of any commercial or financial relationships that could be construed as a potential conflict of interest.
