# Supplementary material for: Cultivar Variation in Hormonal Balance Is a Significant Determinant of Disease Susceptibility to Xanthomonas campestris pv. campestris in Brassica napus
Source: Front Plant Sci. 2017 Dec 12;8:2121. doi: 10.3389/fpls.2017.02121 (PMC5732936; doi:10.3389/fpls.2017.02121)
Supplement: Supplementary file 1 [file Table_1.DOCX]

| **Target gene** | **GenBank Number** | **Forward sequence (5'-3' )** | **Reverse sequence (5'-3' )** |
| --- | --- | --- | --- |
| *PDF 1.2* | AY884023.1 | TGTTTTTGCTGCTTTTGGTG | TCGAATGCACTGATTCTTGC |
| *MYC2* | XM_013880351.1 | ACCAAACGTCTCGAAAATGG | TGTCAACGAGCAAGAGGATG |
| *NPR1* | EF613226.1 | TGAGAACATTGCCAAGCAAG | CAACAGCAAAATGGAGAGCA |
| *TGA1* | XM_013787637.1 | AAGAAGCTTCCACGTCCAGA | TCTATGCCATTGCCAACGTA |
| *CHS* | AF076333.1 | GTCCCTGGACTCATCTCCAA | TACCGTACTCGCTCAACACG |
| *ANR* | XM_013835886.1 | ATCAAACCAGCGGTACAAGG | ACCCCAGTTAAACGGCTTCT |
| *F5H* | DQ679758.1 | TAGACCGTCCAGGTTTTTGG | TGGTTTCATGCCATCAGGTA |
| *ACTIN* | AF111812 | GATTCCGTTGCCCTGAAGTA | GCGACCACCTTGATCTTCAT |

**Supplementary Table S1**. Specific primers used for qRT-PCR.

All primers were designed directly from sequences in the public database.
